# Evaluation of reverse transcription-loop-mediated isothermal amplification for rapid detection of SARS-CoV-2

**DOI:** 10.1038/s41598-021-03623-y

**Published:** 2021-12-20

**Authors:** Willi Quino, Diana Flores-León, Junior Caro-Castro, Carmen V. Hurtado, Iris Silva, Ronnie G. Gavilan

**Affiliations:** 1 Instituto Nacional de Salud, Lima, Perú; 2 Escuela Profesional de Medicina Humana, Universidad Privada San Juan Bautista, Lima, Perú

**Keywords:** Microbiology, Molecular biology

## Abstract

The main strategy for response and control of COVID-19 demands the use of rapid, accurate diagnostic tests aimed at the first point of health care. During the emergency, an increase in asymptomatic and symptomatic cases results in a great demand for molecular tests, which is promoting the development and application of rapid diagnostic technologies. In this study, we describe the development and evaluation of RT-LAMP to detect SARS-CoV-2 based on three genes (*ORF1ab*, *M* and *N* genes) in monoplex and triplex format. RT-LAMP assays were compared with the gold standard method RT-qPCR. The triplex format (*RdRp*, *M* and *N* genes) allowed obtaining comparable results with de RT-qPCR (*RdRp* and *E* genes), presented a sensitivity of 98.9% and a specificity of 97.9%, opening the opportunity to apply this method to detect SARS-CoV-2 at primary health-care centers.

## Introduction

Severe Acute Respiratory Syndrome Coronavirus 2 (SARS-CoV-2) is the causative agent of COVID-19 that emerged in Wuhan, capital city of Hubei province, China in December 2019, and then spread around the world, declared by the World Health Organization (WHO) as a pandemic in March 2020^1–3^. By November 11 2020, more than 51.7 million cases of coronavirus disease 2019 (COVID-19) have been reported in 191 countries, causing deaths of more than 1.2 million people^4^. In Peru, to this date (11/11/2020), more than 925 000 cases and approximately more than 34 000 deaths have been reported as a result of COVID-19^5^.

SARS-CoV-2 has spread primarily through asymptomatic or mildly symptomatic patients^6^. Due to their high rates of mutation and recombination events, coronavirus species can infect many species in both humans and animals^7^. Before the emergence of SARS-CoV-2, six different coronaviruses have been reported to infect humans, including HCoV-229E, HCoV-OC43, HCoV-NL63, HCoV-HKU1, MERS, and SARS-CoV-1. SARS-CoV-2 belongs to the genus β-coronavirus, subgroup 2B, within the *Coronaviridae* family^8^.The SARS-CoV-2 viral genome consists of a highly recombinant 30 Kb positive-sense single-stranded RNA molecule which encodes different genes: The replicase complex (*ORF1ab*), spike protein (*S*), envelope (*E*), membrane (*M*), and nucleoprotein (*N*), in addition to several non-structural genes (*nps*) and accessory proteins^8^. Due to the nature of viral genetics, the *N* gene is the most transcribed and highly conserved within the *Coronaviridae* family being an important target for antigen and antibody diagnosis. RNA-dependent RNA polymerase (*RdRp*), encoded by a fragment of the *ORF1b* region, exhibits a high level of intra-group conservation and, therefore, is an ideal target for its application in diagnosis^9,10^.

The pandemic caused by SARS-CoV-2 threatens global health, so it is urgent to develop rapid and accurate diagnostic methods and their application at the first point-of-care for the effective identification of patients with early infections and their timely treatment, in order to control the spread of this disease^11^.

In light of this global emergency, diagnostic tests based on reverse transcription qPCR (RT-qPCR) have been used; however, this molecular method has some limitations, such as the need for high purity samples, trained personnel, sophisticated facilities for sample processing and the access to expensive laboratory instruments. To address these limitations, the use of loop-mediated isothermal amplification (LAMP) has recently been proposed, which allows direct detection of SARS-CoV-2 RNA^12^, and the result can be directly visualized by color change^13^. Thus, the aim of this study was the development and evaluation of RT-LAMP to detect SARS-CoV-2 RNA virus in nasal and pharyngeal swabs from patients with suspected COVID-19 by comparing the efficacy of 5 different sets of oligonucleotides.

## Methods

### LAMP primers design

The oligonucleotides for the detection of SARS-CoV-2 virus using RT-LAMP were designed to align in the *ORF1ab* to detect the *RdRp* region, as well as to detect the *M* gene and the *N* gene, based on the genome of SARS-CoV-2 Wuhan-Hu-1 virus reference (GenBank Accession: NC_045512.2). Oligonucleotides for RT-LAMP were designed using a LAMP designer software (Premier Biosoft International, USA) and were synthesized by Integrated DNA Technologies (IDT, USA). Three sets of oligonucleotides (*RdRp*, *M* and *N*) were designed, each set consisted of: FIP (Forward Inner Primer), BIP (Backward Inner Primer), F3 (Forward Outer Primer), B3 (Backward Outer Primer), LF (First Forward Loop), LB (First Backward Loop).

### Ethical statement

This study was conducted within the framework of the research project “Platform for the genotyping of SARS-CoV-2 virus” which was approved by the Institutional Committee of Research and Ethics (IRB) of the Instituto Nacional de Salud of Peru (D.R.N°191-2020-OGITT/INS). All procedures and methods were performed in accordance with ethical standards of the Declaration of Helsinki or comparable relevant guidelines and regulation. The approval of an informed consent was waived due to the retrospective nature of this study by the Institutional review board of the Instituto Nacional de Salud of Peru and in accordance with the national legislation and the institutional requirements for Public Health Surveillance.

### Clinical samples

A total of 329 nasal and pharyngeal swab samples were selected within the framework of SARS-CoV-2 National Surveillance of the Instituto Nacional de Salud (INS) of Peru. These samples were sent to the INS for molecular diagnosis by RT-qPCR and genotyping.

### Viral RNA extraction

Viral RNA from clinical samples was extracted using GenUP Total RNA Kit (Biotechrabbit, Germany), following the manufacturer's recommendations. The concentration and quality of the extracted RNA were determined by the DS-11 FX spectrophotometer/fluorometer (DeNovix) and Qubit 3.0 (Invitrogen, Germany).

### RT-qPCR

The samples were analyzed using the SuperScript III One-Step RT-qPCR System with Platinum Taq DNA Polymerase (Invitrogen, Germany), following the manufacturer's recommendations. To prepare the RT-qPCR reaction mix, a real-time reverse transcription enzyme, a primer mix composed by two sets of SARS-CoV-2 oligonucleotides/probes and a set for the human gene (*GAPDH*) were used. To detect the SARS-CoV-2 virus, a reaction mixture with a total volume of 20 μL was prepared: containing a mix of oligonucleotides (0.8 µM *RdRp* gene, 0.4 µM*E* gene, 0.2 µM GAPDH gene; probe: 0.2 uM*RdRp*_SARSr_P1/E_Sarbeco_P and 0.1 GAPDH_P), 0.05 U/μL of Platinum Taq Mix enzyme, 3.2 mM of magnesium sulfate and 5 μL of RNA to each reaction mix. The RT-qPCR conditions were: Reverse transcription: 50 °C for 15 min, Initial denaturation: 95 °C for 2 min, followed by 45 cycles: Denaturation: 95 °C for 15 s, Annealing: 58 °C for 30 s. The Rotor Gene Q Qiagen thermal cycler was used for amplification. The acquisition was carried out in the hybridization step in the green (*RdRp* gene), yellow (*E* gene) and orange (*GAPDH* gene) channel. The presence of SARS-CoV-2 virus was evidenced by the identification of an amplification curve with Ct values  < 40 in the *E*, *RdRp* and human genes mix^14^. When the amplification curves had Ct values > 40 is considered negative.

### RT-LAMP in RNA samples from patients with clinically suspected COVID-19

Five RT-LAMP assays were performed with each sample from the patients considered in this study, using four sets of oligonucleotides that amplify a fragment of *nsp3* (non-structural protein 3—*ORF1a*), *RdRp* (RNA dependent RNA polymerase—*ORF1b*), *M, N* genes, and in the triplex format (*RdRp, M* and *N* mix).

RT-LAMP assays were developed with the WarmStart Colorimetric LAMP 2X Master Mix - M1800 (DNA and RNA) (New England Biolabs, USA)^15^, using a mixture of RT-LAMP oligonucleotides at a final concentration of 1.6 µM FIP and (BIP); 0.2 µMof F3 and B3 primers; 0.4 μM of LF and LB. A reaction mixes with a total volume of 25 μL was prepared: containing 12.5 μl of WarmStart Colorimetric LAMP 2X Master Mix, 2.5 μl of LAMP primer mix, 5 μl of RNase-free molecular grade water (Sigma) and 5 μl of RNA. Positive and negative controls were established according to the manufacturer's recommendations. The reaction was established at 65 °C for 45 min in a Thermo Block (Thermo Scientific Inc, USA). The same procedure was developed with each LAMP sets.

### Limit of detection of 2019-nCoV_*RdRp* positive control RT-LAMP

The limit of detection (LoD) of the RT-LAMP to detect SARS-CoV-2 virus was carried out in triplicate using the positive control 2019-nCoV_RdRp (*ORF1ab*) (Integrated DNA Technologies), which contains the envelope gene and a portion of the RNA-dependent RNA polymerase (*RdRp*) of 2019-nCoV, which has been synthesized at a concentration of 200,000 copies/µL. The predetermined copy numbers of the biochemically synthesized RNA were serially diluted ten-fold from 10^6^ copies to 10^–1^ copies of the target gene per reaction. RT-qPCR to detect the *RdRp* gene was performed using the same dilutions of the positive control.

### Limit of detection of clinical samples using RT-LAMP

The limit of detection for *RdRp, M, N, nsp3* genes and the triplex format (*RdRp, M, N*) was also evaluated in triplicate using a sample confirmed as positive by RT-qPCR for the *RdRp* gene. The RNA concentration of the sample was 47.17 ng/µL, making serial dilutions from 10^0^ to 10^–5^.

### Statistical analysis

The sensitivity and specificity of RT-LAMP was estimated using RT-qPCR as the reference standard with a 95% confidence interval (95% CI). The concordance of the results obtained by RT-qPCR and each of the RT-LAMP assays based on Cohen's kappa statistic were obtained using Stata v15.0 statistical program (Stata Corporation, USA). The sensitivity, specificity and linearity graphs were constructed with GraphPad Prism v7 (GraphPad Inc., USA).

The Spearman's Rank correlation coefficient was used to determine the strength of a link between RT-qPCR results and RT-LAMP evaluated regions (SI. 1).

## Results

### Primer evaluation-RT-LAMP

**Figure 1:**
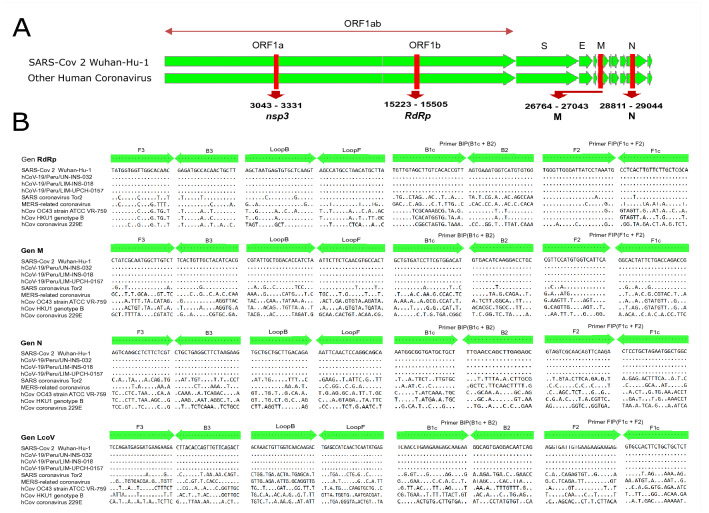
In silico analysis of the oligonucleotide alignment for the RT-LAMP assay included in the study. (**A**) Genetic map of the complete genome of SARS-CoV-2 Wuhan-Hu-1. (**B**) Alignment of the RT-LAMP designed oligonucleotides with the sequences of human coronaviruses: SARS-CoV-2 coronavirus, SARS coronavirus Tor2, MERS-related coronavirus, hCov OC43, hCov HKU1 and hCov 229E. *NoD* No detected, *NTC* Not template control, *MM* master mix.

The oligonucleotides designed in this study were evaluated with the free NCBI Primer-BLAST tool. In this analysis, a set of oligonucleotides that align in the *Orf1a* region was also included to detect a fragment of the *nps3* gene developed by Lamb et al.^16^ (Table 1). The sequences of the designed oligonucleotides were compared with the sequences of human SARS-CoV-2 virus genomes and other coronaviruses described in the NCBI and GISAID: SARS coronavirus Tor2, complete genome (GBA: NC_004718.3); Middle East respiratory syndrome-related coronavirus isolate HCoV-EMC/2012 complete genome (GBA: NC_019843.3), Human coronavirus OC43 strain ATCC VR-759, complete genome (GBA: NC_006213.1); Human coronavirus HKU1, complete genome (GBA: NC_006577.2); Human coronavirus 229E, complete genome (GBA: NC_002645.1); and three genomes described in GISAID: hCoV-19/Peru/UN-INS-032/2020 (GISAID Accession: EPI_ISL_547948); hCoV-19/Peru/LIM-N18/2020 (GISAID Accession: EPI_ISL_490975); hCoV-19/Peru/LIM-UPCH 0157/2020 (GISAID Accession: EPI_ISL_568550). Based on the *in-silico* analysis of the sequences of the primers, a high specificity was observed, aligning 100% with the reference SARS-CoV-2 genome sequences and those genotypes circulating in Peru (Fig. 1).

**Table 1:** RT-LAMP primers for the detection of SARS-CoV-2.

Gene	Primer	Sequence	Reference
Orf1b gene (*RdRp*)	*RdRp*_F3	TATGGTGGTTGGCACAAC	This study
	*RdRp*_B3	AAGCAGTTGTGGCATCTC	
	*RdRp*_FIP	TGCGAGCAAGAACAAGTGAGGTGGGTTGGGATTATCCTAAATG	
	*RdRp*_BIP	TGTTGTAGCTTGTCACACCGTTCCACACATGACCATTTCACT	
	*RdRp*_LoopF	TAAGCATGTTAGGCATGGCT	
	*RdRp*_LoopB	AGCTAATGAGTGTGCTCAAGT	
*M* gene	*M*_F3	CTATCGCAATGGCTTGTCT	This study
	*M*_B3	CGTGATGTAGCAACAGTGA	
	*M*_FIP	CGGTCTGGTCAGAATAGTGCCCGTTCCATGTGGTCATTCA	
	*M*_BIP	GCTGTGATCCTTCGTGGACATGCAGGTCCTTGATGTCAC	
	*M*_LoopF	AGTGGCACGTTGAGAAGAAT	
	*M*_LoopB	CGTATTGCTGGACACCATCTA	
*N* gene	*N*_F3	AGTCAAGCCTCTTCTCGT	This study
	*N*_B3	CTTCTTAGAAGCCTCAGCAG	
	*N*_FIP	GCCAGCCATTCTAGCAGGAGGTAGTCGCAACAGTTCAAGA	
	*N*_BIP	AATGGCGGTGATGCTGCTGCTCTCAAGCTGGTTCAA	
	*N*_LoopF	TGCTGCCTGGAGTTGAATT	
	*N*_LoopB	TTGCTGCTGCTTGACAGA	
Orf1a gene (*nsp3*)	LCoV_F3	TCCAGATGAGGATGAAGAAGA	Lamb, et al.^16^
	LCoV_B3	AGTCTGAACAACTGGTGTAAG	
	LCoV_FIP	AGAGCAGCAGAAGTGGCACAGGTGATTGTGAAGAAGAAGAG	
	LCoV_BIP	TCAACCTGAAGAAGAGCAAGAACTGATTGTCCTCACTGCC	
	LCoV_LoopF	CTCATATTGAGTTGATGGCTCA	
	LCoV_LoopB	ACAAACTGTTGGTCAACAAGAC

### Limit of detection of 2019-nCoV_*RdRp* positive control RT-LAMP

**Figure 2:**
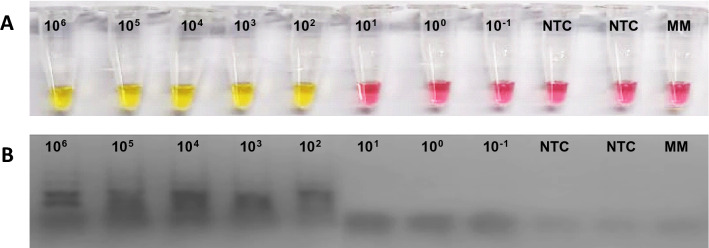
(**A**) Limit of detection of 2019-nCoV_RdRp positive control RT-LAMP. (**B**) Products of RT LAMP in agarose gel match the detection limit.

The limit of detection of RT-qPCR and RT-LAMP assays for the detection of the *RdRp* gene were compared. For the RT-qPCR, the standard curve was linear and a correlation coefficient (R = 0.99325), a slope of − 3.199 and an efficiency of 1.05 were obtained. The LoD of the RT-qPCR was one logarithmic unit higher (1000 copies/reaction) compared to the RT-LAMP (Table 2). Positive RT-LAMP reactions were seen visually as yellow, while negative reactions remained pink viewed with the naked eye (Fig. 2A). The limit of detection was confirmed by a gel electrophoresis of the RT-LAMP products (Fig. 2B).

**Table 2:** Limits of detection of RT-qPCR and RT-LAMP assays for the detection of the *RdRp* gene.

N°	N° of copies	RT-LAMP	RT-qPCR (Ct value)
1	10^6^ copies	Positive	20.19
2	10^5^ copies	Positive	24.47
3	10^4^ copies	Positive	28.93
4	10^3^ copies	Positive	30.94
5	10^2^ copies	Positive	ND
6	10^1^ copies	Negative	ND
7	10^0^ copies	Negative	ND
8	10^–1^ copies	Negative	ND
9	NTC (Negative control)	Negative	ND
10	NTC (Negative control)	Negative	ND
11	Master Mix	Negative	ND

*NoD* No detected, *NTC* not template control.

### Limit of detection of clinical samples using RT-LAMP

**Figure 3:**
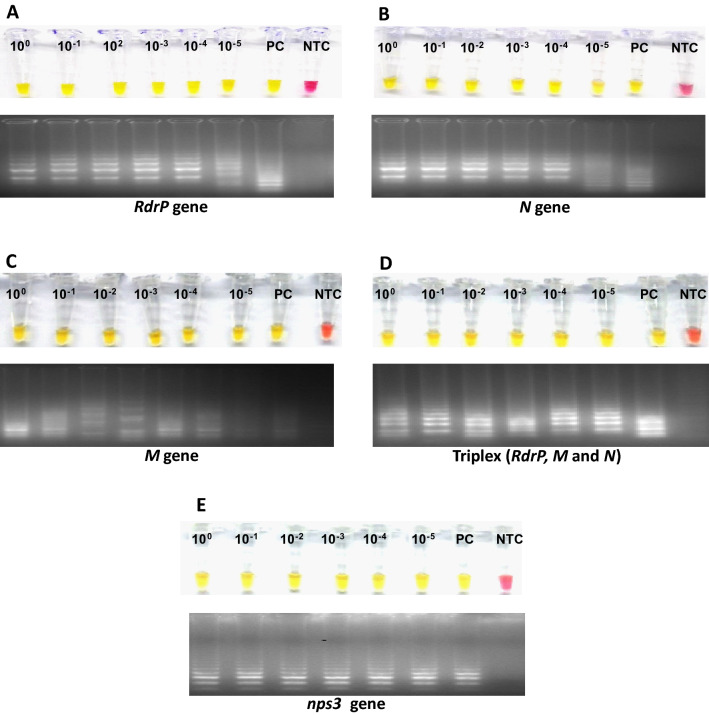
(**A**) Limit of detection (LoD) of sample 1 for the detection of the *RdRp* gene, (**B**) LoD of sample 1 for the detection of the *N* gene, (**C**) LoD of sample 1 for the detection of the *M* gene, (**D**) LoD of Sample 1 for the detection of Triplex (*RdRp*, *M*, *N* genes), (**E**) LoD of sample 1 for the detection of the *nsp3* gene.

The concentration of the evaluated sample was 48.17 ng/µL. Based on the serial dilutions from 10^0^–10^–5^, the limit of detection for the *RdRp, N, M*, the triplex format (*RdRp, M, N* genes) and *nsp3* genes was up to 10^–5^ dilution (Fig. 3).

### RT-qPCR and RT-LAMP of clinical samples

A total of 329 nasal and pharyngeal swabs from patients with clinically suspected COVID-19 from the diagnosis of the SARS-CoV-2 virus from the National Reference Laboratory for Respiratory Viruses were evaluated. A comparative evaluation of RT-qPCR and RT-LAMP was performed. The RT-qPCR for the *RdRp* gene detected 85 positives and 244 negatives; while for the detection of the *E* gene, 83 positives and 246 negatives were detected (SI. 2).

**Figure 4:**
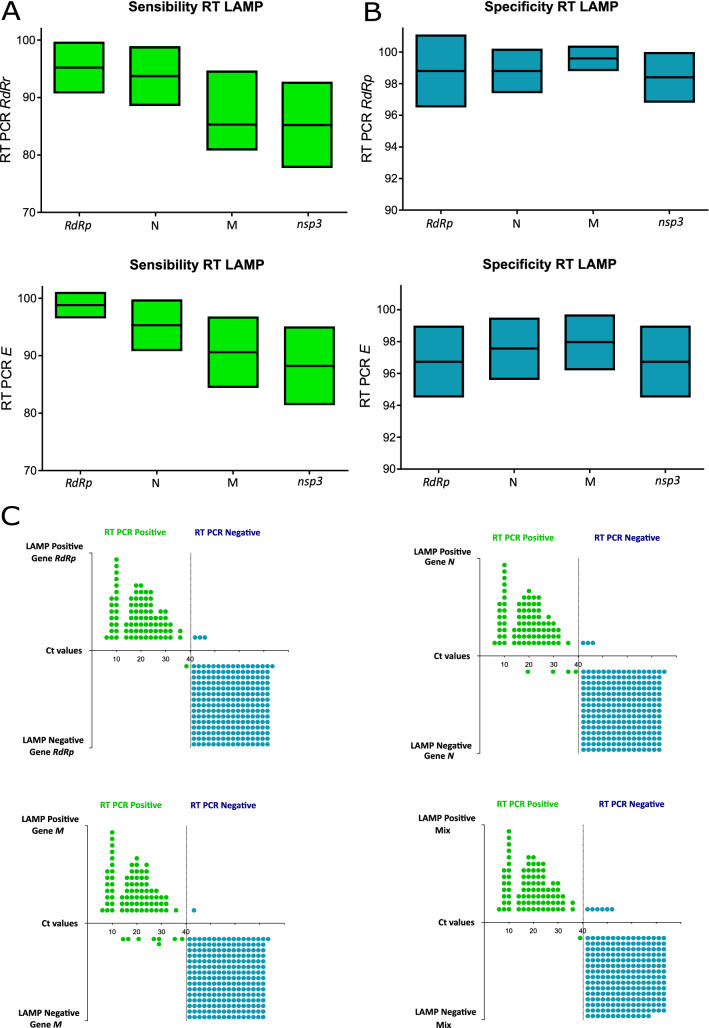
(**A**) Sensitivity and Specificity of RT-LAMP vs RT-qPCR-*RdRp*. (**B**) Sensitivity and Specificity of RT-LAMP vs RT-qPCR-*E*. (**C**) Linearity chart comparing the LAMP positive/negative samples and their detection based on the *RdRp* gene-based qRT-qPCR.

The results of RT-LAMP of *RdRp, M, N*, *nsp3* genes and the triplex format (*RdRp*, *M*, *N*) were compared with the results of RT-qPCR (*RdRp* gene and *E* gene). RT-qPCR based on the *RdRp* gene detected a total of 85 positives and 244 negatives in a cohort of 329 patients. In the comparative analysis of RT-qPCR (*RdRp*) with RT-LAMP based on four molecular detections and a mix of genes, RT-LAMP assays based on the amplification of the triplex format (*RdRp*, *M*, *N*, *nsp3*) presented a sensitivity of 98.9%and a specificity of 97.9%, while the comparative analysis with the *RdRp* gene presented a sensitivity of 98.8% and a specificity of 98.8%,the comparison with the *N* gene presented a sensitivity of 95.3% and a specificity of 98.8%; the comparison with the *M* gene obtained a sensitivity of 90.6% and a specificity of 99.6%; and the comparison with the *nsp3* gene presented a sensitivity of 88.2% and a specificity of 98.4% (Fig. 4A).

The RT-qPCR based on the *E* gene detected a similar number of positives (n = 83) and negatives (n = 246). In the evaluation of the RT-qPCR of the *E* gene, in comparison with the four genes evaluated of the RT-LAMP and a mix of genes, it was determined that the amplification of the triplex format (*RdRp, M, N, nsp3*) presented a sensitivity of 96.4% and a specificity of 95.5%, while the comparative analysis with the *RdRp* gene obtained a sensitivity of 95.2% and a specificity 96.7%; the evaluation with the *N* gene presented a sensitivity of 94% and a specificity of 97.6%; while with the *M* gene obtained a sensitivity of 88% and a specificity of 98%; and the comparative analysis with the *nsp3* gene presented a sensitivity of 85.5% and a specificity of 96.7% (Fig. 4B). In the comparative evaluation of triplex RT-LAMP (*RdRp, M* and *N* genes) with RT-qPCR based on the amplification of *RdRp, N* and *GAPH* genes, no false negatives were observed, reaching a specificity of 100% and a sensitivity of 91.9%, being the positive predictive value of 100% and negative predictive value of 96.6% (Fig. 4B).

In this comparative evaluation, the RT-qPCR directed to the *RdRp* and *E* genes for the identification of the presence of SARS-CoV2 virus RNA, the positive result was considered only if a Ct value ≤ 40 was detected by the RT-qPCR (*RdRp* and *E*).

From the concordance analysis of RT-qPCR results compared with all the genes of RT-LAMP, it is evidenced that there is a high concordance of 0.9685 between the RT-qPCR (*RdRp*) and the RT-LAMP (*RdRp*) (Table 3).

**Table 3:** Level of agreement of RT-qPCR results compared to all RT-LAMP results.

	RT-LAMP	Sensibility (IC 95%)	Specificity (IC 95%)	Positive predictive value (IC 95%)	Negative predictive value (IC 95%)
*RdRp* gene RT-qPCR	*RdRp*	98.8% (96.5%, 101.1%)	98.8% (97.4%, 100.2%)	96.6% (92.7%, 100.4%)	99.6% (98.8%, 100.4%)
	*M*	90.6% (84.4%, 96.8%)	99.6% (98.8%, 100.4%)	98.7% (96.2%, 101.2%)	96.8% (94.6%, 99.0%)
	*N*	95.3% (90.8%, 99.8%)	98.8% (97.4%, 100.2%)	96.4% (92.5%, 100.4%)	98.4% (96.8%, 100.0%)
	nsp3	88.2% (81.4%, 95.1%)	98.4% (96.8%, 100.0%)	94.9% (90.1%, 99.8%)	96.0% (93.6%, 98.4%)
	Triplex	98.9% (93.8% 100%)	97.9% (95.2%, 99.3%)	94.5 (87.6%, 98.2%)	99.6% (97.7%, 100%)
*E* gene RT-qPCR	*RdRp*	95.2% (90.6%, 99.8%)	96.7% (94.5%, 99.0%)	90.8% (84.7%, 96.9%)	90.8% (84.7%, 96.9%)
	*M*	88.0% (80.9%, 95.0%)	98.0% (96.2%, 99.7%)	93.6% (88.2%, 99.0%)	96.0% (93.6%, 98.4%)
	*N*	94.0% (88.9%, 99.1%)	97.6% (95.6%, 99.5%)	92.9% (87.3%, 98.4%)	98.0% (96.2%, 99.7%)
	nsp3	85.5% (78.0%, 93.1%)	96.7% (94.5%, 99.0%)	89.9% (83.2%, 96.5%)	95.2% (92.6%, 97.8%)
	Triplex	96.4% (89.8% 99.2%)	95.5% (92.1%, 97.7%)	87.9% (79.4%, 93.2%)	98.7% (96.4%, 99.7%)

Now, the findings of RT-LAMP based on four molecular detections, in direct correlation with the standard RT-qPCR Ct values of the *RdRp* gene were described. Tracing the positives and negatives results of RT-LAMP based on *RdRp* gene against the linearity of Ct values, it was detected 84 true positives (VP), three false positives (FP), 241 true negatives (VN), and one false negative (FN). RT-LAMP analysis based on *N* gene revealed 81 VP, 03 FP, 241 VN and 04 FN; In contrast using the *M* gene, 77 VP, 01 FP, 243 VN and 08 FN were observed, while the triplex format (*RdRp, M* and *N*) determined 91 VP, 230 VN and 08 FN (Fig. 4C).

## Discussion

Since the beginning of the pandemic caused by the SARS-CoV-2 virus^17^, it has been observed that there is a gap in the availability of molecular tests for the detection of SARS-CoV-2 based on real-time RT-qPCR. Timely and accurate laboratory diagnosis of suspected COVID-19 patients is critical during the COVID-19 pandemic response^18^. This study demonstrates the potential use of a rapid RT-LAMP-based method for the detection of SARS-CoV-2 with high specificity and sensitivity. RT-LAMP has been selected because it provides a rapid and reliable diagnostic alternative for the detection of infectious agents in settings with limited resources such as dengue virus, Zika, chikungunya, influenza, yellow fever among other viruses^19–21^.

Although *ORF1ab* is the confirmatory target gene with the highest specificity, it is considered less sensitive than other clinicallyapplicable targets^22–24^. Although diagnostic assays can be designed in the most conserved region of the viral genome, most of the RT-qPCR and RT-LAMP routinely applied target the *ORF1ab*, *RdRp, S, E* and *N* genes due to their high level of transcription and abundance of expression compared to other SARS-CoV-2 genes^25^, so the design of our oligonucleotides were directed towards these targets.

The high sensitivity and specificity of RT-LAMP obtained in this study is noteworthy, particularly since it is based on the comparison with the reference method RT-qPCR (*RdRp* and *E*) and the amplification of multiple genes of RT-LAMP. These findings have great importance, both clinically and epidemiologically due to the high cases of symptomatic and asymptomatic patients of COVID-19^26,27^, which explain the necessity of the development and application of a portable rapid diagnostic test with good sensitivity and specificity to identify these cases.

In this RT-LAMP optimization assays, the LoD was one logarithmic unit lower (1000 copies/reaction) than the RT-qPCR, when using the positive control 2019-nCoV_*RdRp* (*ORF1ab*) (Table 2). This result showed RT-LAMP assays are more sensitive than RT-qPCR. Several studies using the protocol developed by Corman et al.^14^ reported RT-qPCR assays could not detect most samples with Ct > 30. This group of samples is usually subject to wrong diagnostic treatment or to no treatment at all, spreading the disease to others^28^. Previous studies indicate that Ct > 30 values showed patients without infective capacity, but there is not a real consensus^29^. Additionally, RT-LAMP assays previously reported reaffirm being more sensitive than RT-qPCR, detecting a smaller number of copies, including samples with Ct values between 31–35. For example, Bharda et al. reported that the use of a set of six primers for RT-LAMP can detect 100 to 1000 copies of SARS-CoV-2 genomic RNA^30^. Similarly, Chan et al.^31^ described a RT-LAMP with a LoD of 11.2 RNA copies per reaction using in vitro RNA transcripts, while Yan et al*.*^32^ adapted the *ORF1ab* gene to develop a RT-LAMP with a limit of detection of 20 copies per reaction. Most of these diagnostic tests have a high level of sensitivity, specificity, and repeatability; however, these mainly lack clinical validation^33^.

In this study, we performing a RT-LAMP using five sets of oligonucleotides, the triplex format (*RdRp, M, N*) was the one which presented a sensitivity of 98.9% and a specificity of 97.9% when was compared to the RT-qPCR (*RdRp* gene and *E* gene), since the *ORF1ab* gene is very specific and the *N* gene is very sensitive in addition to the *M* gene, data very similar to the reported by Yang et al*.*^34^ with a sensitivity of 99%. Likewise, the highly conserved *RdRp* gene presented a sensitivity and a specificity of 98.8%, very similar to those reported by Kitagawa et al*.*^35^, who obtained a sensitivity and a specificity of 100% and 97% respectively, as well as the sensitivity and specificity for the *N* and *M* genes: 95.3% and 98.8%; 90.6% and 99.6% respectively, data very similar to that reported by Jiang et al.^36^ with a sensitivity and specificity of 91.4% and 99.5%, respectively, while the sensitivity and specificity for the *nsp3* gene was 88.2% and 98.4% respectively, data very similar to the reported by Hu et al.^37^.

There are several reports about RT-LAMP monoplex assays, obtaining different values of sensitivity and specificity. For example, Yu et al.^38^ and Dao Thi et al.^39^ reported sensitivities of 97.6%, and 97.5% respectively, which were comparable to results of this study. However, the study of Escalante-Maldonado et al.^40^ obtained lower sensitivity (87.4%) than this study. In contrast, RT-LAMP triplex assays were reported based on different sets of primers, having advantages and disadvantages when they are compared to this study. Yamazaki et al.^41^, using primers for *ORF1ab, S* and *ORF7a* regions, obtaining a sensitivity of 82.6% and specificity of 100% and showing the poorest sensibility compared to nasal-pharyngeal samples. Another, study reported by Sherrill-Mix et al.^42^ using primers E1 (*E* protein), As1e (*ORF1a*), and Penn (*ORF1ab*), getting a LoD greater than 100 copies/reaction that was comparable to this study results. Juscamayta-Lopez et al.^43^ included the *RdRp, M* and *N* genes for their triplex RT-LAMP with sensitivity and specificity values of 100.0% and 98.6% that were comparable to this study. However, the method needs to use a turbidimeter equipment to measure the color change during the assay which is difficult to apply in low-resource settings.

An important limitation for RT-LAMP assay is the RNA extraction and purification, which is essential during the use of nasal-pharyngeal or saliva samples without RNA purification. Some studies overcome these difficulties by applying different strategies such as Wei et al.^44^ that designed a method without the need for prior RNA extraction, processing samples inside transport media in 30 min and obtaining a high sensitivity. However, the sensitivity is strongly affected by processing samples after 30 min. In this study, we performed RT-LAMP assays including samples stored to − 80 °C from a few days to several months, and the sensitivity was not affected.

In conclusion, the RT-LAMP using the triplex format (*RdRp*, *M* and *N* genes) was the one which obtained best numbers of sensitivity (98.9%) and specificity (97.7%), obtaining comparable results with RT-qPCR to detect SARS-CoV-2 RNA virus in nasal and pharyngeal swabs, opening the opportunity to perform this method from patients with suspected COVID-19 at primary health-care centers.

## Supplementary Information

Supplementary Information.
