# Supplementary material for: Evaluation of reverse transcription-loop-mediated isothermal amplification for rapid detection of SARS-CoV-2
Source: Sci Rep. 2021 Dec 20;11:24234. doi: 10.1038/s41598-021-03623-y (PMC8688429; doi:10.1038/s41598-021-03623-y)

Evaluation of Reverse Transcription-Loop Mediated Isothermal Amplification for rapid detection of SARS-CoV-2

Willi Quino[1], Diana Flores[1], Junior Caro-Castro[1], Carmen V Hurtado[1], Iris Silva[1], Ronnie G. Gavilan[1]*

[1]Instituto Nacional de Salud. Lima, Perú. ***email:** rgavilan@ins.gob.pe

Supplementary information 1. Spearman's Rank correlation coefficient between RT-qPCR results and RT-LAMP evaluated regions.

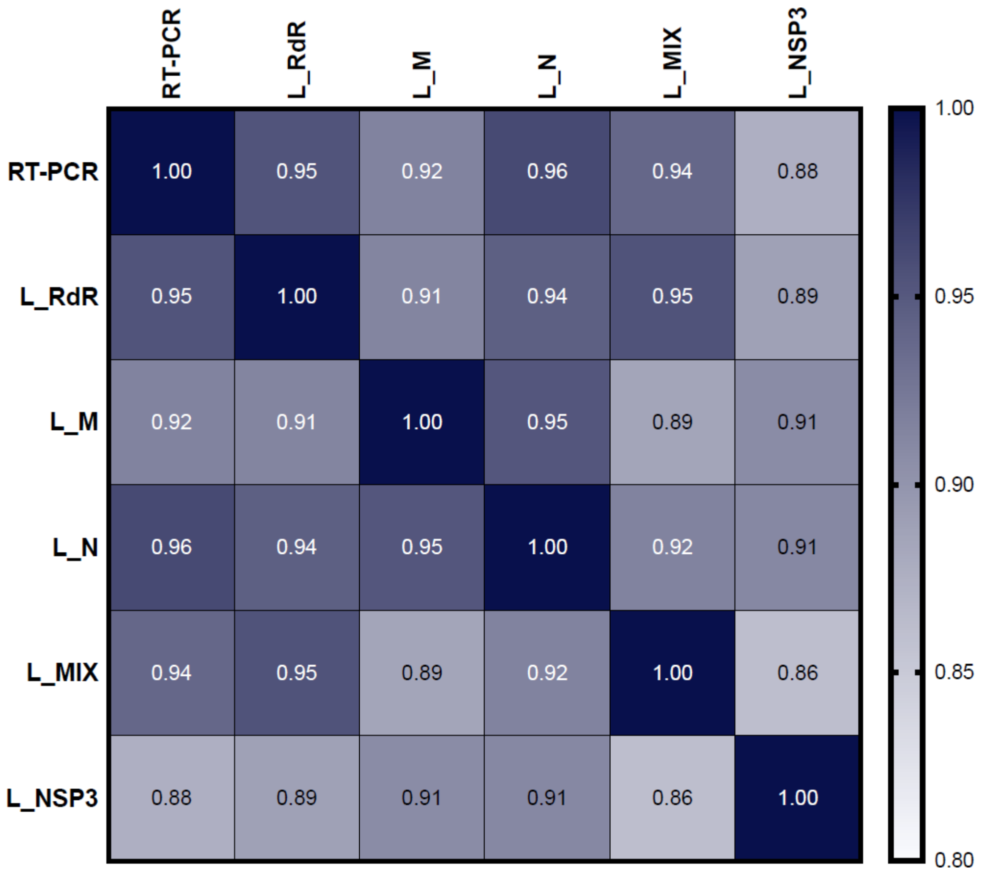

Supplementary information 2. RT-qPCR and RT-LAMP of 329 clinical samples.

N	RT-qPCR						RT-LAMP					
	RdRP gene		*E* gene		*GADPH* gene		*RdRp* gene	*M* gene	*N* gene	Triplex (*RdRp, M, N*)	*nsp3* gene	Human control
	CT	Interpretation	CT	Interpretation	CT	Interpretation						
1	Negative	Negative	Negative	Negative	25.1	Positive	Negative	Negative	Negative	Negative	Negative	Positive
2	Negative	Negative	Negative	Negative	26.8	Positive	Negative	Negative	Negative	Negative	Positive	Positive
3	17.6	Positive	17.87	Positive	23.2	Positive	Positive	Positive	Positive	Positive	Positive	Positive
4	Negative	Negative	Negative	Negative	21.26	Positive	Negative	Negative	Negative	Negative	Negative	Positive
5	Negative	Negative	Negative	Negative	28.18	Positive	Negative	Negative	Negative	Positive	Positive	Positive
6	Negative	Negative	Negative	Negative	26.11	Positive	Negative	Negative	Negative	Positive	Negative	Positive
7	19.05	Positive	24.46	Positive	23.95	Positive	Positive	Positive	Positive	Positive	Positive	Positive
8	29.17	Positive	Negative	Negative	27.48	Positive	Positive	Negative	Positive	Positive	Positive	Positive
9	28.98	Positive	18.48	Positive	26.15	Positive	Positive	Positive	Positive	Positive	Positive	Positive
10	18.64	Positive	25.22	Positive	24.34	Positive	Positive	Positive	Positive	Positive	Positive	Positive
11	13.67	Positive	13.9	Positive	22.2	Positive	Positive	Positive	Positive	Positive	Positive	Positive
12	22.03	Positive	21.09	Positive	23.87	Positive	Positive	Positive	Positive	Positive	Positive	Positive
13	22.29	Positive	22.85	Positive	24.52	Positive	Positive	Positive	Positive	Positive	Positive	Positive
14	16.61	Positive	15.9	Positive	26.64	Positive	Positive	Positive	Positive	Positive	Positive	Positive
15	Negative	Negative	Negative	Negative	26.86	Positive	Negative	Negative	Negative	Negative	Negative	Positive
16	28.47	Positive	Negative	Negative	26.47	Positive	Positive	Positive	Positive	Positive	Positive	Positive
17	26.73	Positive	Negative	Negative	26.3	Positive	Positive	Positive	Positive	Positive	Positive	Positive
18	16.91	Positive	16.73	Positive	29.09	Positive	Positive	Positive	Positive	Positive	Positive	Positive
19	15.56	Positive	Negative	Negative	25.7	Positive	Positive	Positive	Positive	Positive	Positive	Positive
20	13	Positive	Negative	Negative	24.9	Positive	Positive	Positive	Positive	Positive	Positive	Positive
21	31.71	Positive	29.22	Positive	30.72	Positive	Positive	Positive	Positive	Positive	Positive	Positive
22	14.07	Positive	Negative	Negative	26.61	Positive	Positive	Positive	Positive	Positive	Negative	Positive
23	18.51	Positive	25.43	Positive	29.85	Positive	Positive	Positive	Positive	Positive	Positive	Positive
24	30.98	Positive	27.72	Positive	26.37	Positive	Positive	Positive	Positive	Positive	Positive	Positive
25	Negative	Negative	Negative	Negative	27.14	Positive	Negative	Negative	Negative	Positive	Negative	Positive
26	19.66	Positive	17.74	Positive	22.64	Positive	Positive	Positive	Positive	Positive	Positive	Positive
27	36.33	Positive	33.46	Positive	30.39	Positive	Positive	Negative	Negative	Positive	Negative	Positive
28	23.37	Positive	25.43	Positive	29.62	Positive	Positive	Positive	Positive	Positive	Positive	Positive
29	30.78	Positive	28.32	Positive	29.72	Positive	Positive	Positive	Positive	Positive	Positive	Positive
30	23.76	Positive	22.06	Positive	28.76	Positive	Positive	Positive	Positive	Positive	Positive	Positive
31	20.94	Positive	18.64	Positive	27.82	Positive	Positive	Positive	Positive	Positive	Positive	Positive
32	31.43	Positive	30.57	Positive	29.44	Positive	Positive	Positive	Positive	Positive	Positive	Positive
33	26.31	Positive	24.68	Positive	28.03	Positive	Positive	Positive	Positive	Positive	Positive	Positive
34	28.25	Positive	25.36	Positive	26.25	Positive	Positive	Positive	Positive	Positive	Positive	Positive
35	20.76	Positive	18.63	Positive	29.39	Positive	Positive	Positive	Positive	Positive	Positive	Positive
36	24.84	Positive	22.97	Positive	28.67	Positive	Positive	Positive	Positive	Positive	Positive	Positive
37	35.04	Positive	28.65	Positive	25.01	Positive	Positive	Positive	Positive	Positive	Positive	Positive
38	23.06	Positive	33.36	Positive	27.74	Positive	Positive	Positive	Positive	Positive	Negative	Positive
39	26.38	Positive	24.02	Positive	29.67	Positive	Positive	Positive	Positive	Positive	Positive	Positive

40	28.35	Positive	26.66	Positive	29.86	Positive	Positive	Positive	Positive	Positive	Positive	Positive
41	23.24	Positive	20.56	Positive	25.99	Positive	Positive	Positive	Positive	Positive	Positive	Positive
42	24.18	Positive	21.76	Positive	25.63	Positive	Positive	Positive	Positive	Positive	Positive	Positive
43	29.89	Positive	27.27	Positive	29.45	Positive	Positive	Positive	Positive	Positive	Positive	Positive
44	24.15	Positive	21.78	Positive	26.2	Positive	Positive	Positive	Positive	Positive	Positive	Positive
45	32.73	Positive	28.35	Positive	29.26	Positive	Positive	Positive	Positive	Positive	Positive	Positive
46	24.56	Positive	22.07	Positive	27.39	Positive	Positive	Positive	Positive	Positive	Positive	Positive
47	21.28	Positive	19.03	Positive	28.55	Positive	Positive	Positive	Positive	Positive	Positive	Positive
48	18.29	Positive	16.5	Positive	24.62	Positive	Positive	Positive	Positive	Positive	Positive	Positive
49	21.21	Positive	19.6	Positive	25.12	Positive	Positive	Positive	Positive	Positive	Positive	Positive
50	21.05	Positive	18.71	Positive	28.54	Positive	Positive	Positive	Positive	Positive	Positive	Positive
51	21.43	Positive	18.32	Positive	28.98	Positive	Positive	Positive	Positive	Positive	Positive	Positive
52	Negative	Negative	Negative	Negative	29.18	Positive	Positive	Negative	Negative	Positive	Negative	Positive
53	22.34	Positive	31.3	Positive	25.41	Positive	Positive	Positive	Positive	Positive	Positive	Positive
54	Negative	Negative	Negative	Negative	26.52	Positive	Positive	Negative	Negative	Negative	Positive	Positive
55	Negative	Negative	Negative	Negative	25.32	Positive	Negative	Negative	Negative	Negative	Negative	Positive
56	20.03	Positive	27.82	Positive	23.46	Positive	Positive	Positive	Positive	Positive	Positive	Positive
57	19.04	Positive	24.39	Positive	27.82	Positive	Positive	Positive	Positive	Positive	Positive	Positive
58	16.8	Positive	18.84	Positive	25.71	Positive	Positive	Positive	Positive	Positive	Positive	Positive
59	18.11	Positive	16.28	Positive	27.38	Positive	Positive	Positive	Positive	Positive	Positive	Positive
60	19.28	Positive	24.04	Positive	25.5	Positive	Positive	Positive	Positive	Positive	Positive	Positive
61	16.63	Positive	27	Positive	25.35	Positive	Positive	Positive	Positive	Positive	Positive	Positive
62	19.34	Positive	24.76	Positive	31.02	Positive	Positive	Positive	Positive	Positive	Positive	Positive
63	20.2	Positive	29.19	Positive	24.46	Positive	Positive	Positive	Positive	Positive	Positive	Positive
64	10.11	Positive	30.93	Positive	24.61	Positive	Positive	Positive	Positive	Positive	Positive	Positive
65	9.55	Positive	23.86	Positive	22.6	Positive	Positive	Positive	Positive	Positive	Positive	Positive
66	8.88	Positive	19.1	Positive	19.87	Positive	Positive	Positive	Positive	Positive	Positive	Positive
67	9.37	Positive	22.52	Positive	20.49	Positive	Positive	Positive	Positive	Positive	Positive	Positive
68	8.65	Positive	20.78	Positive	22.25	Positive	Positive	Positive	Positive	Positive	Positive	Positive
69	8.87	Positive	19.1	Positive	21.65	Positive	Positive	Positive	Positive	Positive	Positive	Positive
70	8.85	Positive	20.01	Positive	23.3	Positive	Positive	Positive	Positive	Positive	Positive	Positive
71	8.06	Positive	25.01	Positive	23.56	Positive	Positive	Positive	Positive	Positive	Positive	Positive
72	Negative	Negative	Negative	Negative	22.69	Positive	Negative	Negative	Negative	Positive	Negative	Positive
73	9.65	Positive	26.48	Positive	22.21	Positive	Positive	Positive	Positive	Positive	Positive	Positive
74	9.1	Positive	18.6	Positive	23.8	Positive	Positive	Positive	Positive	Positive	Positive	Positive
75	9.15	Positive	21.16	Positive	21.82	Positive	Positive	Positive	Positive	Positive	Positive	Positive
76	8.74	Positive	18.09	Positive	24	Positive	Positive	Positive	Positive	Positive	Positive	Positive
77	Negative	Negative	29.37	Positive	24.24	Positive	Positive	Negative	Positive	Positive	Negative	Positive
78	Negative	Negative	28.55	Positive	24.51	Positive	Negative	Positive	Positive	Negative	Negative	Positive
79	18.49	Positive	29.33	Positive	23.16	Positive	Positive	Positive	Positive	Positive	Positive	Positive
80	Negative	Negative	Negative	Negative	25.15	Positive	Negative	Negative	Negative	Negative	Negative	Positive
81	8.38	Positive	24.16	Positive	23.88	Positive	Positive	Positive	Positive	Positive	Positive	Positive
82	Negative	Negative	20.22	Positive	24.56	Positive	Negative	Negative	Negative	Positive	Negative	Positive
83	Negative	Negative	31.19	Positive	25.52	Positive	Negative	Negative	Positive	Negative	Positive	Positive

84	10.32	Positive	21.01	Positive	24.51	Positive	Positive	Positive	Positive	Positive	Positive	Positive
85	9.93	Positive	21.53	Positive	23.53	Positive	Positive	Positive	Positive	Positive	Positive	Positive
86	9.15	Positive	15.18	Positive	23.31	Positive	Positive	Positive	Positive	Positive	Positive	Positive
87	Negative	Negative	19.63	Positive	18.96	Positive	Negative	Negative	Negative	Negative	Negative	Positive
88	9.29	Positive	18.82	Positive	23.71	Positive	Positive	Positive	Positive	Positive	Positive	Positive
89	6.72	Positive	10.86	Positive	21.27	Positive	Positive	Positive	Positive	Positive	Positive	Positive
90	9.58	Positive	24.43	Positive	23.97	Positive	Positive	Positive	Positive	Positive	Positive	Positive
91	9.34	Positive	19.97	Positive	24.73	Positive	Positive	Positive	Positive	Positive	Positive	Positive
92	9.5	Positive	20.53	Positive	24.01	Positive	Positive	Positive	Positive	Positive	Positive	Positive
93	Negative	Negative	Negative	Negative	25.12	Positive	Negative	Negative	Negative	Negative	Negative	Positive
94	Negative	Negative	Negative	Negative	26.52	Positive	Negative	Negative	Negative	Negative	Negative	Positive
95	Negative	Negative	Negative	Negative	22.3	Positive	Negative	Negative	Negative	Negative	Negative	Positive
96	Negative	Negative	Negative	Negative	24.26	Positive	Negative	Negative	Negative	Negative	Negative	Positive
97	Negative	Negative	Negative	Negative	27.18	Positive	Negative	Negative	Negative	Negative	Negative	Positive
98	Negative	Negative	Negative	Negative	28.11	Positive	Negative	Negative	Negative	Negative	Negative	Positive
99	Negative	Negative	Negative	Negative	24.95	Positive	Negative	Negative	Negative	Negative	Negative	Positive
100	Negative	Negative	Negative	Negative	26.48	Positive	Negative	Negative	Negative	Negative	Negative	Positive
101	Negative	Negative	Negative	Negative	24.15	Positive	Negative	Negative	Negative	Negative	Negative	Positive
102	Negative	Negative	Negative	Negative	28.34	Positive	Negative	Negative	Negative	Negative	Negative	Positive
103	Negative	Negative	Negative	Negative	21.23	Positive	Negative	Negative	Negative	Negative	Negative	Positive
104	Negative	Negative	Negative	Negative	22.87	Positive	Negative	Negative	Negative	Negative	Negative	Positive
105	Negative	Negative	Negative	Negative	26.52	Positive	Negative	Negative	Negative	Negative	Negative	Positive
106	Negative	Negative	Negative	Negative	24.64	Positive	Negative	Negative	Negative	Negative	Negative	Positive
107	Negative	Negative	Negative	Negative	28.86	Positive	Negative	Negative	Negative	Negative	Negative	Positive
108	Negative	Negative	Negative	Negative	23.47	Positive	Negative	Negative	Negative	Negative	Negative	Positive
109	Negative	Negative	Negative	Negative	27.32	Positive	Negative	Negative	Negative	Negative	Negative	Positive
110	40	Positive	Negative	Negative	30.09	Positive	Negative	Negative	Negative	Negative	Negative	Positive
111	Negative	Negative	Negative	Negative	24.73	Positive	Negative	Negative	Negative	Negative	Negative	Positive
112	Negative	Negative	Negative	Negative	24.21	Positive	Negative	Negative	Negative	Negative	Negative	Positive
113	Negative	Negative	Negative	Negative	29.11	Positive	Negative	Negative	Negative	Negative	Negative	Positive
114	Negative	Negative	Negative	Negative	24.43	Positive	Negative	Negative	Negative	Negative	Negative	Positive
115	Negative	Negative	Negative	Negative	30.45	Positive	Negative	Negative	Negative	Negative	Negative	Positive
116	Negative	Negative	Negative	Negative	25.24	Positive	Negative	Negative	Negative	Negative	Negative	Positive
117	Negative	Negative	Negative	Negative	26.18	Positive	Negative	Negative	Negative	Negative	Negative	Positive
118	Negative	Negative	Negative	Negative	23.45	Positive	Negative	Negative	Negative	Negative	Negative	Positive
119	Negative	Negative	Negative	Negative	29.4	Positive	Negative	Negative	Negative	Negative	Negative	Positive
120	Negative	Negative	Negative	Negative	28.23	Positive	Negative	Negative	Negative	Negative	Negative	Positive
121	Negative	Negative	Negative	Negative	27.24	Positive	Negative	Negative	Negative	Negative	Negative	Positive
122	Negative	Negative	Negative	Negative	27.45	Positive	Negative	Negative	Negative	Negative	Negative	Positive
123	Negative	Negative	Negative	Negative	26.1	Positive	Negative	Negative	Negative	Negative	Negative	Positive
124	Negative	Negative	Negative	Negative	30.22	Positive	Negative	Negative	Negative	Negative	Negative	Positive
125	Negative	Negative	Negative	Negative	27.24	Positive	Negative	Negative	Negative	Negative	Negative	Positive
126	Negative	Negative	Negative	Negative	27.23	Positive	Negative	Negative	Negative	Negative	Negative	Positive
127	Negative	Negative	Negative	Negative	30.58	Positive	Negative	Negative	Negative	Negative	Negative	Positive

128	Negative	Negative	Negative	Negative	27.24	Positive	Negative	Negative	Negative	Negative	Negative	Positive
129	Negative	Negative	Negative	Negative	26.1	Positive	Negative	Negative	Negative	Negative	Negative	Positive
130	Negative	Negative	Negative	Negative	28.56	Positive	Negative	Negative	Negative	Negative	Negative	Positive
131	Negative	Negative	Negative	Negative	30.22	Positive	Negative	Negative	Negative	Negative	Negative	Positive
132	Negative	Negative	Negative	Negative	30.23	Positive	Negative	Negative	Negative	Negative	Negative	Positive
133	Negative	Negative	Negative	Negative	19.48	Positive	Negative	Negative	Negative	Negative	Negative	Positive
134	Negative	Negative	Negative	Negative	24.26	Positive	Negative	Negative	Negative	Negative	Negative	Positive
135	Negative	Negative	Negative	Negative	30.18	Positive	Negative	Negative	Negative	Negative	Negative	Positive
136	Negative	Negative	Negative	Negative	25.4	Positive	Negative	Negative	Negative	Negative	Negative	Positive
137	Negative	Negative	Negative	Negative	30.1	Positive	Negative	Negative	Negative	Negative	Negative	Positive
138	Negative	Negative	Negative	Negative	28.26	Positive	Negative	Negative	Negative	Negative	Negative	Positive
139	Negative	Negative	Negative	Negative	29.54	Positive	Negative	Negative	Negative	Negative	Negative	Positive
140	Negative	Negative	Negative	Negative	25.23	Positive	Negative	Negative	Negative	Negative	Negative	Positive
141	Negative	Negative	Negative	Negative	26.3	Positive	Negative	Negative	Negative	Negative	Negative	Positive
142	Negative	Negative	Negative	Negative	29.2	Positive	Negative	Negative	Negative	Negative	Negative	Positive
143	Negative	Negative	Negative	Negative	29.4	Positive	Negative	Negative	Negative	Negative	Negative	Positive
144	Negative	Negative	Negative	Negative	28.4	Positive	Negative	Negative	Negative	Negative	Negative	Positive
145	Negative	Negative	Negative	Negative	26.62	Positive	Negative	Negative	Negative	Negative	Negative	Positive
146	Negative	Negative	Negative	Negative	25.92	Positive	Negative	Negative	Negative	Negative	Negative	Positive
147	Negative	Negative	Negative	Negative	23.95	Positive	Negative	Negative	Negative	Negative	Negative	Positive
148	Negative	Negative	Negative	Negative	24.66	Positive	Negative	Negative	Negative	Negative	Negative	Positive
149	Negative	Negative	Negative	Negative	58.21	Positive	Negative	Negative	Negative	Negative	Negative	Positive
150	Negative	Negative	Negative	Negative	23.15	Positive	Negative	Negative	Negative	Negative	Negative	Positive
151	Negative	Negative	Negative	Negative	26.98	Positive	Negative	Negative	Negative	Negative	Negative	Positive
152	Negative	Negative	Negative	Negative	26.1	Positive	Negative	Negative	Negative	Negative	Negative	Positive
153	Negative	Negative	Negative	Negative	26.3	Positive	Negative	Negative	Negative	Negative	Negative	Positive
154	Negative	Negative	Negative	Negative	30.68	Positive	Negative	Negative	Negative	Negative	Negative	Positive
155	Negative	Negative	Negative	Negative	23.9	Positive	Negative	Negative	Negative	Negative	Negative	Positive
156	Negative	Negative	Negative	Negative	23.5	Positive	Negative	Negative	Negative	Negative	Negative	Positive
157	Negative	Negative	Negative	Negative	23.6	Positive	Negative	Negative	Negative	Negative	Negative	Positive
158	Negative	Negative	Negative	Negative	20.16	Positive	Negative	Negative	Negative	Negative	Negative	Positive
159	Negative	Negative	Negative	Negative	21.62	Positive	Negative	Negative	Negative	Negative	Negative	Positive
160	Negative	Negative	Negative	Negative	22.64	Positive	Negative	Negative	Negative	Negative	Negative	Positive
161	Negative	Negative	Negative	Negative	22.68	Positive	Negative	Negative	Negative	Negative	Negative	Positive
162	Negative	Negative	Negative	Negative	20.14	Positive	Negative	Negative	Negative	Negative	Negative	Positive
163	Negative	Negative	Negative	Negative	22.14	Positive	Negative	Negative	Negative	Negative	Negative	Positive
164	Negative	Negative	Negative	Negative	23.88	Positive	Negative	Negative	Negative	Negative	Negative	Positive
165	Negative	Negative	Negative	Negative	20.22	Positive	Negative	Negative	Negative	Negative	Negative	Positive
166	Negative	Negative	Negative	Negative	22.9	Positive	Negative	Negative	Negative	Negative	Negative	Positive
167	Negative	Negative	Negative	Negative	24.6	Positive	Negative	Negative	Negative	Negative	Negative	Positive
168	Negative	Negative	Negative	Negative	26.48	Positive	Negative	Negative	Negative	Negative	Negative	Positive
169	Negative	Negative	Negative	Negative	25	Positive	Negative	Negative	Negative	Negative	Negative	Positive
170	Negative	Negative	Negative	Negative	25.48	Positive	Negative	Negative	Negative	Negative	Negative	Positive
171	Negative	Negative	Negative	Negative	22.96	Positive	Negative	Negative	Negative	Negative	Negative	Positive

172	Negative	Negative	Negative	Negative	26.58	Positive	Negative	Negative	Negative	Negative	Negative	Positive
173	Negative	Negative	Negative	Negative	24.22	Positive	Negative	Negative	Negative	Negative	Negative	Positive
174	Negative	Negative	Negative	Negative	25.46	Positive	Negative	Negative	Negative	Negative	Negative	Positive
175	Negative	Negative	Negative	Negative	26.24	Positive	Negative	Negative	Negative	Negative	Negative	Positive
176	Negative	Negative	Negative	Negative	23.47	Positive	Negative	Negative	Negative	Negative	Negative	Positive
177	Negative	Negative	Negative	Negative	22.32	Positive	Negative	Negative	Negative	Negative	Negative	Positive
178	Negative	Negative	Negative	Negative	24.1	Positive	Negative	Negative	Negative	Negative	Negative	Positive
179	Negative	Negative	Negative	Negative	19.2	Positive	Negative	Negative	Negative	Negative	Negative	Positive
180	Negative	Negative	Negative	Negative	22.75	Positive	Negative	Negative	Negative	Negative	Negative	Positive
181	Negative	Negative	Negative	Negative	20.18	Positive	Negative	Negative	Negative	Negative	Negative	Positive
182	Negative	Negative	Negative	Negative	26.2	Positive	Negative	Negative	Negative	Negative	Negative	Positive
183	Negative	Negative	Negative	Negative	23.6	Positive	Negative	Negative	Negative	Negative	Negative	Positive
184	Negative	Negative	Negative	Negative	20.8	Positive	Negative	Negative	Negative	Negative	Negative	Positive
185	Negative	Negative	Negative	Negative	31.2	Positive	Negative	Negative	Negative	Negative	Negative	Positive
186	Negative	Negative	Negative	Negative	26.4	Positive	Negative	Negative	Negative	Negative	Negative	Positive
187	Negative	Negative	Negative	Negative	24.8	Positive	Negative	Negative	Negative	Negative	Negative	Positive
188	Negative	Negative	Negative	Negative	20.14	Positive	Negative	Negative	Negative	Negative	Negative	Positive
189	Negative	Negative	Negative	Negative	19.2	Positive	Negative	Negative	Negative	Negative	Negative	Positive
190	Negative	Negative	Negative	Negative	24.68	Positive	Negative	Negative	Negative	Negative	Negative	Positive
191	Negative	Negative	Negative	Negative	28.98	Positive	Negative	Negative	Negative	Negative	Negative	Positive
192	Negative	Negative	Negative	Negative	30.14	Positive	Negative	Negative	Negative	Negative	Negative	Positive
193	Negative	Negative	Negative	Negative	26.52	Positive	Negative	Negative	Negative	Negative	Negative	Positive
194	Negative	Negative	Negative	Negative	24.18	Positive	Negative	Negative	Negative	Negative	Negative	Positive
195	Negative	Negative	Negative	Negative	26.32	Positive	Negative	Negative	Negative	Negative	Negative	Positive
196	Negative	Negative	Negative	Negative	20.18	Positive	Negative	Negative	Negative	Negative	Negative	Positive
197	Negative	Negative	Negative	Negative	22.84	Positive	Negative	Negative	Negative	Negative	Negative	Positive
198	Negative	Negative	Negative	Negative	22.24	Positive	Negative	Negative	Negative	Negative	Negative	Positive
199	Negative	Negative	Negative	Negative	24.58	Positive	Negative	Negative	Negative	Negative	Negative	Positive
200	Negative	Negative	Negative	Negative	24.32	Positive	Negative	Negative	Negative	Negative	Negative	Positive
201	Negative	Negative	Negative	Negative	28.44	Positive	Negative	Negative	Negative	Negative	Negative	Positive
202	Negative	Negative	Negative	Negative	28.68	Positive	Negative	Negative	Negative	Negative	Negative	Positive
203	Negative	Negative	Negative	Negative	30.12	Positive	Negative	Negative	Negative	Negative	Negative	Positive
204	Negative	Negative	Negative	Negative	26.48	Positive	Negative	Negative	Negative	Negative	Negative	Positive
205	Negative	Negative	Negative	Negative	26.48	Positive	Negative	Negative	Negative	Negative	Negative	Positive
206	Negative	Negative	Negative	Negative	20.18	Positive	Negative	Negative	Negative	Negative	Negative	Positive
207	Negative	Negative	Negative	Negative	24.8	Positive	Negative	Negative	Negative	Negative	Negative	Positive
208	Negative	Negative	Negative	Negative	24	Positive	Negative	Negative	Negative	Negative	Negative	Positive
209	Negative	Negative	Negative	Negative	23.14	Positive	Negative	Negative	Negative	Negative	Negative	Positive
210	Negative	Negative	Negative	Negative	22.14	Positive	Negative	Negative	Negative	Negative	Negative	Positive
211	Negative	Negative	Negative	Negative	20.14	Positive	Negative	Negative	Negative	Negative	Negative	Positive
212	Negative	Negative	Negative	Negative	29.56	Positive	Negative	Negative	Negative	Negative	Negative	Positive
213	Negative	Negative	Negative	Negative	28.54	Positive	Negative	Negative	Negative	Negative	Negative	Positive
214	Negative	Negative	Negative	Negative	21.1	Positive	Negative	Negative	Negative	Negative	Negative	Positive
215	Negative	Negative	Negative	Negative	30.1	Positive	Negative	Negative	Negative	Negative	Negative	Positive

216	Negative	Negative	Negative	Negative	28.2	Positive	Negative	Negative	Negative	Negative	Negative	Positive
217	Negative	Negative	Negative	Negative	25.4	Positive	Negative	Negative	Negative	Negative	Negative	Positive
218	Negative	Negative	Negative	Negative	26.12	Positive	Negative	Negative	Negative	Negative	Negative	Positive
219	Negative	Negative	Negative	Negative	24.6	Positive	Negative	Negative	Negative	Negative	Negative	Positive
220	Negative	Negative	Negative	Negative	30.1	Positive	Negative	Negative	Negative	Negative	Negative	Positive
221	Negative	Negative	Negative	Negative	26.4	Positive	Negative	Negative	Negative	Negative	Negative	Positive
222	Negative	Negative	Negative	Negative	24.16	Positive	Negative	Negative	Negative	Negative	Negative	Positive
223	Negative	Negative	Negative	Negative	26.48	Positive	Negative	Negative	Negative	Negative	Negative	Positive
224	Negative	Negative	Negative	Negative	24.12	Positive	Negative	Negative	Negative	Negative	Negative	Positive
225	Negative	Negative	Negative	Negative	26.32	Positive	Negative	Negative	Negative	Negative	Negative	Positive
226	Negative	Negative	Negative	Negative	24.6	Positive	Negative	Negative	Negative	Negative	Negative	Positive
227	Negative	Negative	Negative	Negative	23.14	Positive	Negative	Negative	Negative	Negative	Negative	Positive
228	Negative	Negative	Negative	Negative	24.56	Positive	Negative	Negative	Negative	Negative	Negative	Positive
229	Negative	Negative	Negative	Negative	24.65	Positive	Negative	Negative	Negative	Negative	Negative	Positive
230	Negative	Negative	Negative	Negative	31.25	Positive	Negative	Negative	Negative	Negative	Negative	Positive
231	Negative	Negative	Negative	Negative	28.15	Positive	Negative	Negative	Negative	Negative	Negative	Positive
232	Negative	Negative	Negative	Negative	24.62	Positive	Negative	Negative	Negative	Negative	Negative	Positive
233	Negative	Negative	Negative	Negative	23.14	Positive	Negative	Negative	Negative	Negative	Negative	Positive
234	Negative	Negative	Negative	Negative	29.48	Positive	Negative	Negative	Negative	Negative	Negative	Positive
235	Negative	Negative	Negative	Negative	27.22	Positive	Negative	Negative	Negative	Negative	Negative	Positive
236	Negative	Negative	Negative	Negative	30.8	Positive	Negative	Negative	Negative	Negative	Negative	Positive
237	Negative	Negative	Negative	Negative	24.84	Positive	Negative	Negative	Negative	Negative	Negative	Positive
238	Negative	Negative	Negative	Negative	23.24	Positive	Negative	Negative	Negative	Negative	Negative	Positive
239	Negative	Negative	Negative	Negative	24.26	Positive	Negative	Negative	Negative	Negative	Negative	Positive
240	Negative	Negative	Negative	Negative	20.28	Positive	Negative	Negative	Negative	Negative	Negative	Positive
241	Negative	Negative	Negative	Negative	19.42	Positive	Negative	Negative	Negative	Negative	Negative	Positive
242	Negative	Negative	Negative	Negative	22.3	Positive	Negative	Negative	Negative	Negative	Negative	Positive
243	Negative	Negative	Negative	Negative	27.01	Positive	Negative	Negative	Negative	Negative	Negative	Positive
244	Negative	Negative	Negative	Negative	22.4	Positive	Negative	Negative	Negative	Negative	Negative	Positive
245	Negative	Negative	Negative	Negative	23.21	Positive	Negative	Negative	Negative	Negative	Negative	Positive
246	Negative	Negative	Negative	Negative	24.26	Positive	Negative	Negative	Negative	Negative	Negative	Positive
247	Negative	Negative	Negative	Negative	30.87	Positive	Negative	Negative	Negative	Negative	Negative	Positive
248	Negative	Negative	Negative	Negative	22.88	Positive	Negative	Negative	Negative	Negative	Negative	Positive
249	Negative	Negative	Negative	Negative	30.4	Positive	Negative	Negative	Negative	Negative	Negative	Positive
250	Negative	Negative	Negative	Negative	23	Positive	Negative	Negative	Negative	Negative	Negative	Positive
251	Negative	Negative	Negative	Negative	18	Positive	Negative	Negative	Negative	Negative	Negative	Positive
252	Negative	Negative	Negative	Negative	19.42	Positive	Negative	Negative	Negative	Negative	Negative	Positive
253	Negative	Negative	Negative	Negative	20.65	Positive	Negative	Negative	Negative	Negative	Negative	Positive
254	Negative	Negative	Negative	Negative	20.58	Positive	Negative	Negative	Negative	Negative	Negative	Positive
255	Negative	Negative	Negative	Negative	24.5	Positive	Negative	Negative	Negative	Negative	Negative	Positive
256	Negative	Negative	Negative	Negative	25.6	Positive	Negative	Negative	Negative	Negative	Negative	Positive
257	Negative	Negative	Negative	Negative	23.4	Positive	Negative	Negative	Negative	Negative	Negative	Positive
258	Negative	Negative	Negative	Negative	20.8	Positive	Negative	Negative	Negative	Negative	Negative	Positive
259	Negative	Negative	Negative	Negative	19.4	Positive	Negative	Negative	Negative	Negative	Negative	Positive

260	Negative	Negative	Negative	Negative	18.4	Positive	Negative	Negative	Negative	Negative	Negative	Positive
261	Negative	Negative	Negative	Negative	23.45	Positive	Negative	Negative	Negative	Negative	Negative	Positive
262	Negative	Negative	Negative	Negative	26.48	Positive	Negative	Negative	Negative	Negative	Negative	Positive
263	Negative	Negative	Negative	Negative	29.4	Positive	Negative	Negative	Negative	Negative	Negative	Positive
264	Negative	Negative	Negative	Negative	20.14	Positive	Negative	Negative	Negative	Negative	Negative	Positive
265	Negative	Negative	Negative	Negative	18.22	Positive	Negative	Negative	Negative	Negative	Negative	Positive
266	Negative	Negative	Negative	Negative	20.14	Positive	Negative	Negative	Negative	Negative	Negative	Positive
267	Negative	Negative	Negative	Negative	23.22	Positive	Negative	Negative	Negative	Negative	Negative	Positive
268	Negative	Negative	Negative	Negative	26.8	Positive	Negative	Negative	Negative	Negative	Negative	Positive
269	Negative	Negative	Negative	Negative	23.53	Positive	Negative	Negative	Negative	Negative	Negative	Positive
270	Negative	Negative	Negative	Negative	24.68	Positive	Negative	Negative	Negative	Negative	Negative	Positive
271	Negative	Negative	Negative	Negative	20.23	Positive	Negative	Negative	Negative	Negative	Negative	Positive
272	Negative	Negative	Negative	Negative	19.58	Positive	Negative	Negative	Negative	Negative	Negative	Positive
273	Negative	Negative	Negative	Negative	20.48	Positive	Negative	Negative	Negative	Negative	Negative	Positive
274	Negative	Negative	Negative	Negative	19.56	Positive	Negative	Negative	Negative	Negative	Negative	Positive
275	Negative	Negative	Negative	Negative	22.98	Positive	Negative	Negative	Negative	Negative	Negative	Positive
276	Negative	Negative	Negative	Negative	23.45	Positive	Negative	Negative	Negative	Negative	Negative	Positive
277	Negative	Negative	Negative	Negative	23.48	Positive	Negative	Negative	Negative	Negative	Negative	Positive
278	Negative	Negative	Negative	Negative	18.4	Positive	Negative	Negative	Negative	Negative	Negative	Positive
279	Negative	Negative	Negative	Negative	30.4	Positive	Negative	Negative	Negative	Negative	Negative	Positive
280	Negative	Negative	Negative	Negative	26.45	Positive	Negative	Negative	Negative	Negative	Negative	Positive
281	Negative	Negative	Negative	Negative	31.02	Positive	Negative	Negative	Negative	Negative	Negative	Positive
282	Negative	Negative	Negative	Negative	24.18	Positive	Negative	Negative	Negative	Negative	Negative	Positive
283	Negative	Negative	Negative	Negative	18.4	Positive	Negative	Negative	Negative	Negative	Negative	Positive
284	Negative	Negative	Negative	Negative	19.32	Positive	Negative	Negative	Negative	Negative	Negative	Positive
285	Negative	Negative	Negative	Negative	23.21	Positive	Negative	Negative	Negative	Negative	Negative	Positive
286	Negative	Negative	Negative	Negative	26.23	Positive	Negative	Negative	Negative	Negative	Negative	Positive
287	Negative	Negative	Negative	Negative	28.41	Positive	Negative	Negative	Negative	Negative	Negative	Positive
288	Negative	Negative	Negative	Negative	25.52	Positive	Negative	Negative	Negative	Negative	Negative	Positive
289	Negative	Negative	Negative	Negative	26.48	Positive	Negative	Negative	Negative	Negative	Negative	Positive
290	Negative	Negative	Negative	Negative	26.12	Positive	Negative	Negative	Negative	Negative	Negative	Positive
291	Negative	Negative	Negative	Negative	28.23	Positive	Negative	Negative	Negative	Negative	Negative	Positive
292	Negative	Negative	Negative	Negative	30.14	Positive	Negative	Negative	Negative	Negative	Negative	Positive
293	Negative	Negative	Negative	Negative	28.4	Positive	Negative	Negative	Negative	Negative	Negative	Positive
294	Negative	Negative	Negative	Negative	26.4	Positive	Negative	Negative	Negative	Negative	Negative	Positive
295	Negative	Negative	Negative	Negative	18.49	Positive	Negative	Negative	Negative	Negative	Negative	Positive
296	Negative	Negative	Negative	Negative	19.25	Positive	Negative	Negative	Negative	Negative	Negative	Positive
297	Negative	Negative	Negative	Negative	22.1	Positive	Negative	Negative	Negative	Negative	Negative	Positive
298	Negative	Negative	Negative	Negative	26.8	Positive	Negative	Negative	Negative	Negative	Negative	Positive
299	Negative	Negative	Negative	Negative	26.4	Positive	Negative	Negative	Negative	Negative	Negative	Positive
300	Negative	Negative	Negative	Negative	23.14	Positive	Negative	Negative	Negative	Negative	Negative	Positive
301	Negative	Negative	Negative	Negative	22.1	Positive	Negative	Negative	Negative	Negative	Negative	Positive
302	Negative	Negative	Negative	Negative	19.2	Positive	Negative	Negative	Negative	Negative	Negative	Positive
303	Negative	Negative	Negative	Negative	30.12	Positive	Negative	Negative	Negative	Negative	Negative	Positive

304	Negative	Negative	Negative	Negative	19.23	Positive	Negative	Negative	Negative	Negative	Negative	Positive
305	Negative	Negative	Negative	Negative	26.28	Positive	Negative	Negative	Negative	Negative	Negative	Positive
306	Negative	Negative	Negative	Negative	29.13	Positive	Negative	Negative	Negative	Negative	Negative	Positive
307	Negative	Negative	Negative	Negative	30.2	Positive	Negative	Negative	Negative	Negative	Negative	Positive
308	Negative	Negative	Negative	Negative	19.23	Positive	Negative	Negative	Negative	Negative	Negative	Positive
309	Negative	Negative	Negative	Negative	24.68	Positive	Negative	Negative	Negative	Negative	Negative	Positive
310	Negative	Negative	Negative	Negative	27.32	Positive	Negative	Negative	Negative	Negative	Negative	Positive
311	Negative	Negative	Negative	Negative	26.45	Positive	Negative	Negative	Negative	Negative	Negative	Positive
312	Negative	Negative	Negative	Negative	25.8	Positive	Negative	Negative	Negative	Negative	Negative	Positive
313	Negative	Negative	Negative	Negative	29.18	Positive	Negative	Negative	Negative	Negative	Negative	Positive
314	Negative	Negative	Negative	Negative	28.14	Positive	Negative	Negative	Negative	Negative	Negative	Positive
315	Negative	Negative	Negative	Negative	25.56	Positive	Negative	Negative	Negative	Negative	Negative	Positive
316	Negative	Negative	Negative	Negative	23.24	Positive	Negative	Negative	Negative	Negative	Negative	Positive
317	Negative	Negative	Negative	Negative	22.14	Positive	Negative	Negative	Negative	Negative	Negative	Positive
318	Negative	Negative	Negative	Negative	20.1	Positive	Negative	Negative	Negative	Negative	Negative	Positive
319	Negative	Negative	Negative	Negative	19.4	Positive	Negative	Negative	Negative	Negative	Negative	Positive
320	Negative	Negative	Negative	Negative	18.4	Positive	Negative	Negative	Negative	Negative	Negative	Positive
321	18.6	Positive	24.56	Positive	24.53	Positive	Positive	Positive	Positive	Positive	Positive	Positive
322	26.3	Positive	28.17	Positive	23.88	Positive	Positive	Positive	Positive	Positive	Positive	Positive
323	17.57	Positive	40.67	Positive	26.54	Positive	Positive	Positive	Positive	Positive	Negative	Positive
324	15.79	Positive	25.37	Positive	25.86	Positive	Positive	Positive	Positive	Positive	Negative	Positive
325	30.68	Positive	36.56	Positive	27.1	Positive	Positive	Negative	Negative	Positive	Negative	Positive
326	27.32	Positive	28.43	Positive	25.16	Positive	Positive	Negative	Positive	Positive	Negative	Positive
327	21.92	Positive	33.19	Positive	25.34	Positive	Positive	Negative	Positive	Positive	Negative	Positive
328	18.78	Positive	32.84	Positive	25.07	Positive	Positive	Negative	Negative	Positive	Negative	Positive
329	16.97	Positive	38.07	Positive	28.1	Positive	Positive	Negative	Positive	Positive	Positive	Positive

Supplementary information 3. Full-length gel of Figure 2B.

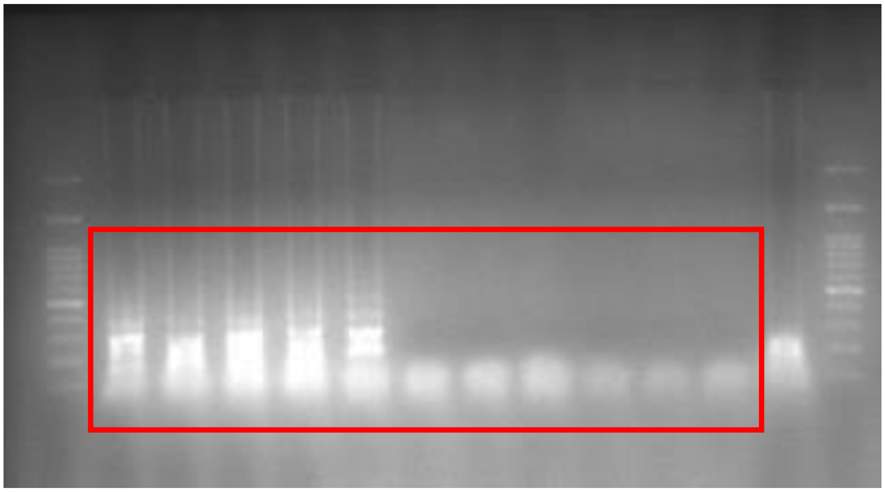

Supplementary information 4. Full-length gel of Figure 3A (left) and 3B (right).

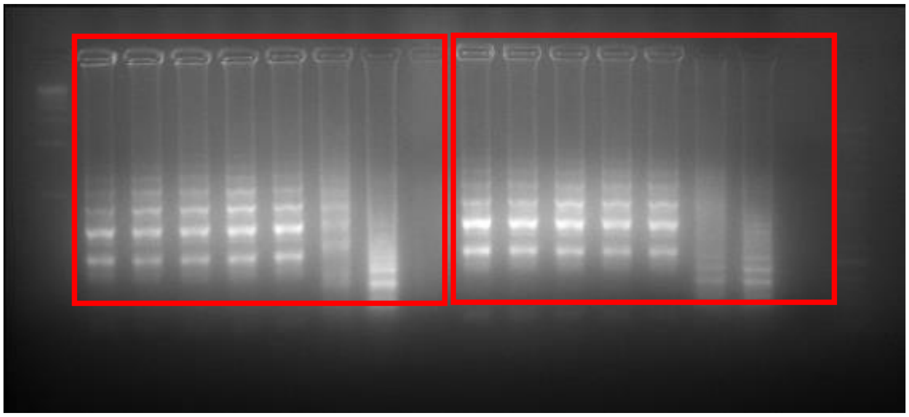

Supplementary information 5. Full-length gel of Figure 3C.

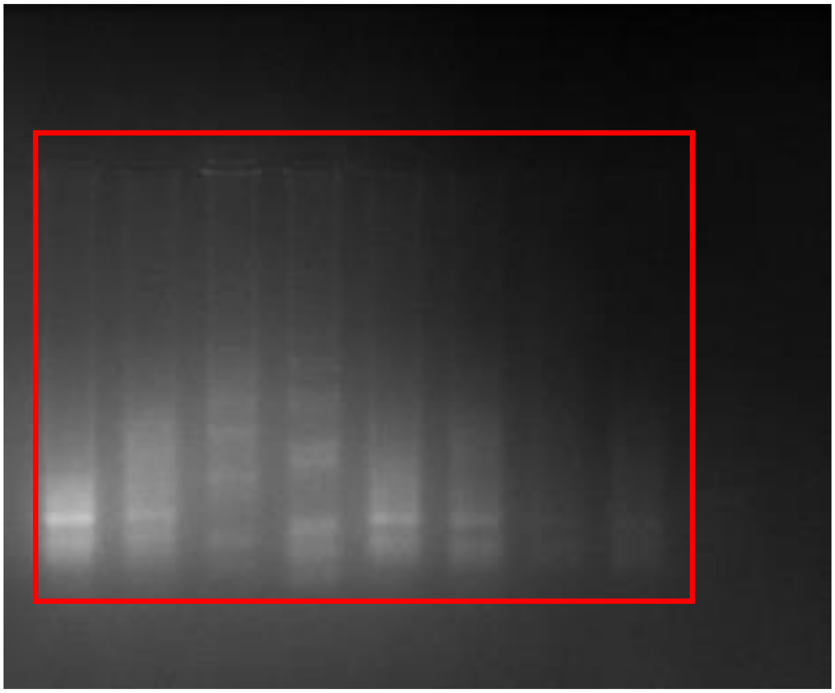

Supplementary information 6. Full-length gel of Figure 3D.

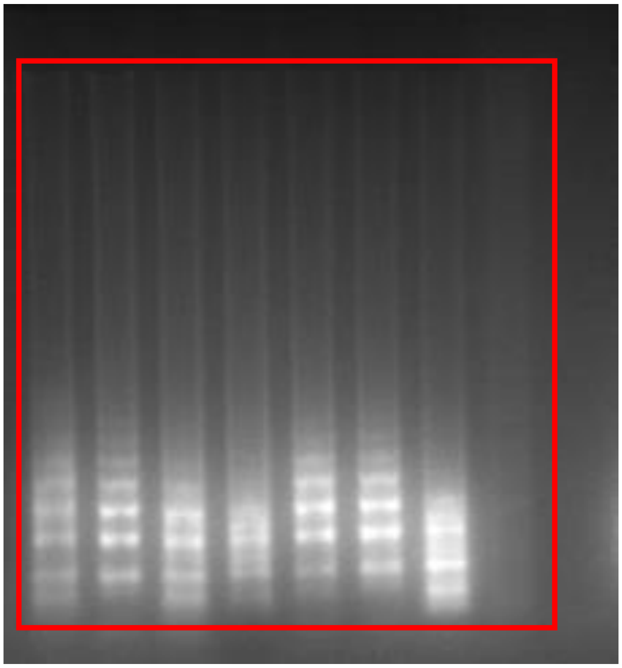

Supplementary information 7. Full-length gel of Figure 3E.

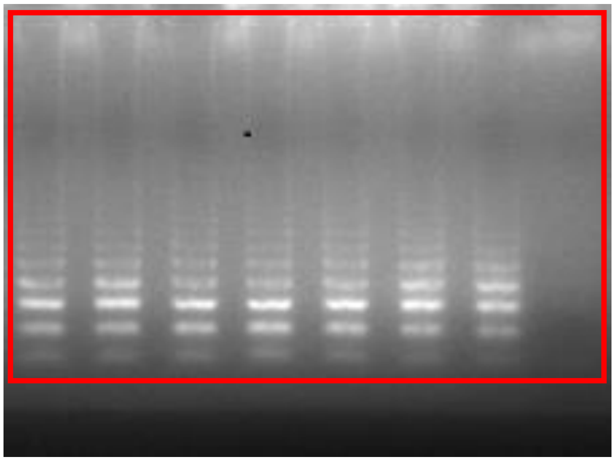

Supplement: Supplementary file 1 — Supplementary Information. [file 41598_2021_3623_MOESM1_ESM.pdf]
